# Epidemic keratoconjunctivitis: efficacy of outbreak management

**DOI:** 10.1007/s00417-021-05344-4

**Published:** 2021-08-18

**Authors:** Cristina Martin, Ursula Löw, Adrien Quintin, Gesine Schießl, Barbara Gärtner, Albert Heim, Berthold Seitz

**Affiliations:** 1 Department of Ophthalmology, Saarland University Medical Center (UKS), Homburg, Saar Germany; 2 Institute of Medical Microbiology and Hygiene, Saarland University, Homburg, Saar Germany; 3 Institute of Virology, Medical Institute of Hannover (MHH), Hannover, Germany

**Keywords:** Epidemic keratoconjunctivitis, EKC outbreak, Hygienic management concept, Basic reproduction number

## Abstract

**Purpose:**

Epidemic keratoconjunctivitis (EKC) is one of the most severe ocular viral infections. The aim of this interruptive time series study was to quantitatively evaluate the effectiveness of a hygienic EKC outbreak management concept developed in our ophthalmological department.

**Methods:**

All patients with suspected EKC in the period from August to November 2018 were included in the study. Data were retrospectively collected from the patient’s medical documents and records. The disease was diagnosed clinically and confirmed by virus detection through polymerase chain reaction (PCR) from conjunctival swabs. With the beginning of the epidemic, an outbreak management plan was implemented to reduce the nosocomial spread.

**Results:**

The outbreak lasted 77 days (20th August 2018 to 4th November 2018) and affected a total of 120 patients. This corresponds to a mean of 1.5 patients per outbreak day. The median age was 58 [1–92] years. Of all patients, 61 (50.8%) were female. Conjunctival swabs were collected in 100/120 (83.3%) cases, the adenovirus being detected in all positive smears (63/63, 100%). The implementation of our outbreak management plan reduced significantly the number of EKC cases per outbreak day and resulted in a reduction of the basic reproduction number by a factor of 2.2.

**Conclusion:**

The detection of EKC together with the immediate implementation of hygienic outbreak measures can significantly reduce the spread of infection. The implementation of a strict outbreak management concept can significantly reduce the number of EKC cases, thus avoiding possible complications and therefore unnecessary health-related costs.


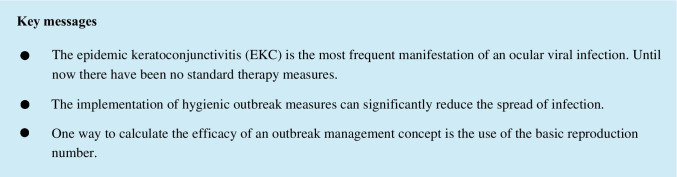


## Introduction

Viruses represent about 80% of all causes of acute conjunctivitis, with epidemic keratoconjunctivitis (EKC) being the most frequent manifestation of an ocular viral infection [1, 2]. EKC is a highly contagious infectious disease,^1^ affecting in Japan alone approximately one million individuals each year [2]. In Germany, a declining trend of EKC outbreaks was reported in the past few years, from 2145 cases in 2012 to just 647 cases in 2019 [3]. However, a considerably higher prevalence can be assumed, because of unreported cases, since only the detection of adenovirus in EKC is notifiable and not every patient with this disease will consult an ophthalmologist or will be tested.

EKC is caused by the human adenovirus (HAdV) which belongs to the genus *Mastadenovirus* of the Adenoviridae family [4]. Meanwhile, 54 different virus serotypes are known, divided into seven species (A–G) [5]. The serotypes 8, 37, 53, 54, and 64 (previously, 19a) of species and HAdV-D are mainly responsible for EKC, while several other types of species HAdV-B and HAdV-E are associated with less severe conjunctivitis and pharyngoconjunctival fever [5].

Adenoviruses are non-enveloped viruses that mainly infect various mucosal membranes including ocular structures [1]. Due to the missing envelope, the adenoviruses are very stable in the environment and highly resistant to commonly used physical agents and chemicals, which may destroy the lipid envelope of other enveloped viruses, contributing even more to their contagiosity [6]. The incubation period ranges from 5 to 12 days [7] with patients being infectious for up to 3 weeks [2]. Most cases are self-limiting with mildly symptomatic subclinical infections [2, 8].

Transmission of adenoviruses occurs either directly through contact with infected persons (e.g., ocular secretions, smearing) or indirectly through contaminated surfaces (such as medical instruments or towels) [2]. Because EKC patients shed high virus loads with ocular secretions, an epidemic outbreak can easily occur [2]. The highly infectious nature of the adenoviral conjunctivitis and its long period of incubation lead to an increased prevalence in the average population, prolonged nosocomial outbreaks, and therefore, considerable medical costs [8].

Therapy of EKC is mostly symptom-oriented since no standardized specific therapy has yet been established [8–10]. Without an effective disease treatment, hygienic measures to prevent EKC outbreaks are of great importance. Good hygiene practices including rigorous disinfection of the hands and instruments [8] and avoiding touching the eyes, nose, and mouth with unwashed hands as well as avoiding contact with acutely infected persons were all described as imperative to prevent EKC spreading [11].

Many studies reported about the necessity of hygienic measures in EKC outbreaks [2, 5]. However, a structured schedule for secondary preventive measures in such cases is still missing. Moreover, none of the studies tried to quantify the efficacy of these hygienic preventive measures in the context of an EKC outbreak.

We hypothesized, in the context of an EKC outbreak, that the implementation of our EKC outbreak management concept will reduce the number of nosocomial EKC cases and contribute to the nosocomial outbreak cessation. Thus, the aim of this hygiene intervention study was to quantitatively evaluate the effectiveness of an easily to be implemented hygienic EKC outbreak management concept developed in our department of ophthalmology.

## Patients and methods

### Study design and population

An interrupted time series analysis of an EKC outbreak between 20th August 2018 and 4th November 2018 was performed in our ophthalmological department. All patients (*N* = 120) who presented with signs and symptoms of EKC within this time period were enrolled in the study. The implementation of the outbreak management concept was prompted by an increasing number of EKC cases. The outbreak management concept protocol and study endpoints were defined in the course of the EKC outbreak. The outbreak was reported in accordance with outbreak reports and intervention studies of nosocomial infection (ORION) recommended criteria [12].

### Definitions

*Suspected EKC cases* were defined as any clinically suspected case of infectious conjunctivitis, characterized by symptoms that included severe foreign body sensation, pain, itching, or photophobia accompanied by tearing or eye discharge.

*Internal EKC cases* included all suspected cases that have had any kind of contact with our ophthalmological department (including medical examinations and operations).

*External EKC cases* included all cases that were not previously examined or treated in our ophthalmological department.

### Data collection

Data were retrospectively collected from patient’s medical records. All data were anonymized and entered in a dedicated electronic file. Before analysis, an independent medical reviewer verified all data for accuracy.

A medical history was taken daily to collect data on new affected patients, number of affected family members, smear results, previous treatments, and examinations in our eye hospital or in an outpatient ophthalmologic department. General information such as age, gender, time of onset, and duration of symptoms was collected.

### Diagnosis

The diagnosis of EKC was suspected when patients presented clinical features including conjunctival redness, chemosis, punctate keratopathy, and subepithelial infiltrates accompanied by symptoms such as severe foreign body sensation, pain, itching, photophobia, tearing, or eye discharge. A conjunctival swab was taken in each suspected EKC case. A presumptive clinical diagnosis of EKC was confirmed when the polymerase chain reaction (PCR) of this conjunctival swab was positive for adenoviruses.

### Outbreak phases and outcomes

The outbreak comprehended 3 epidemiological phases. The first phase started with the first diagnosed case of EKC on 20th August 2018 and ended on 20th September 2018 when the EKC outbreak management was first applied. The second phase began on 20th of September and ended 12 days (maximal incubation time reported for EKC) later on 2nd October 2018. The third phase of the EKC outbreak began on 3rd October 2018 and ended on 4th November 2018. The primary outcome of the study was the number of EKC infections per day of outbreak in each individual outbreak phase. The secondary outcome included basic reproductive number for the first two outbreak phases combined and for the third phase in internal EKC cases.

### Calculation of the basic reproduction number

The appropriate formula to calculate the basic reproduction number R0 for infectious diseases like EKC is [13]

$$\mathrm{R}0=1+\mathrm{r}2\ast \mathrm{t}1\ast \mathrm{t}2+r\ \left(\mathrm{t}1+\mathrm{t}2\right)$$

where *r* is the estimated intrinsic growth rate (estimated from the initial exponential growth phase), t1 is the exposed period (an individual is infected, has no symptoms, and is not infectious), and t2 is the latent infectious period (an individual is infected, has no symptoms, and is infectious). The incubation period represents the duration between infection and the onset of the first symptoms or signs of the disease and can therefore be expressed as exposed time plus latent infectious time.

We defined the initial exponential growth phase between the date of the first internal EKC case and 12 days (maximal reported incubation time) after EKC outbreak management concept implementation.

### EKC outbreak management

**Fig. 1:**
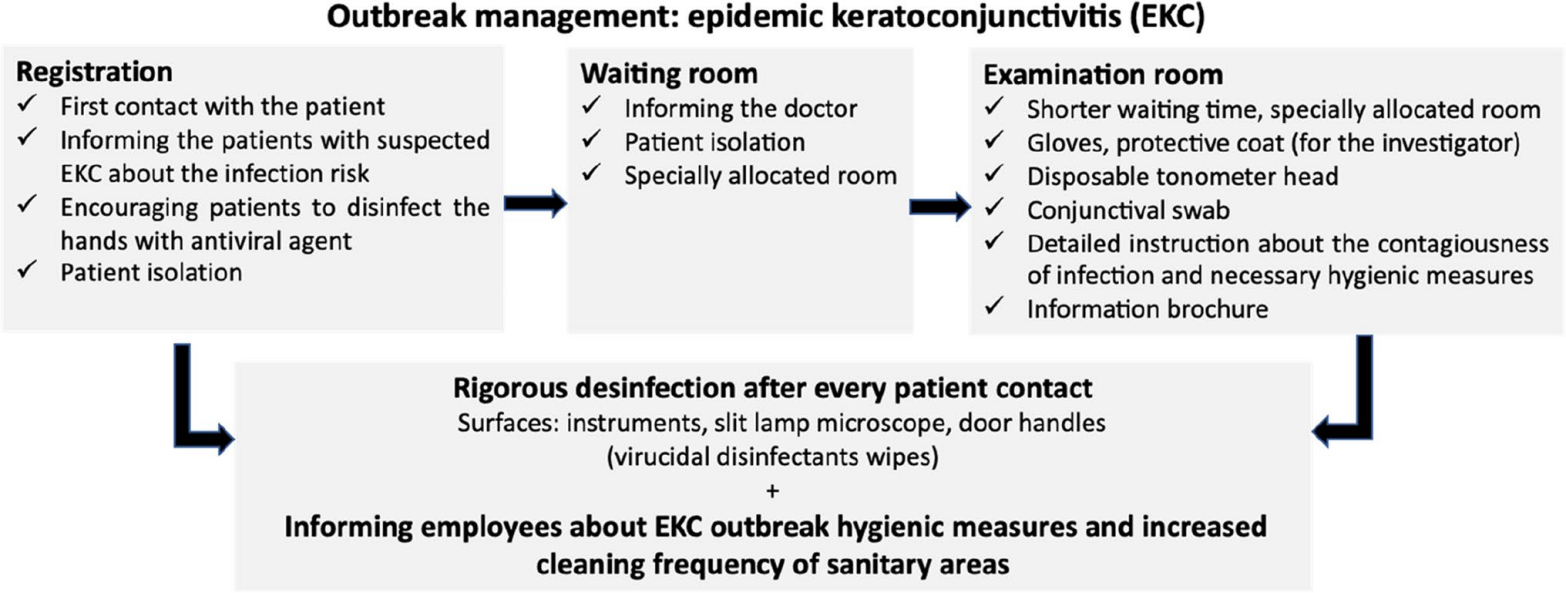
Outbreak management in patients with suspected EKC in the Department of Ophthalmology, Saarland University Medical Center

As the EKC outbreak began, a hygiene concept was put into practice, for both patients and medical staff. Patients were extensively informed about the highly contagious EKC disease and were provided with virucidal hand disinfectants. Every patient with EKC suspicion was isolated and treated separately from other patients in one separated room (general outpatients department room). All medical staff wore disposal medical examination gloves. Hands, medical instruments, and contact surfaces were rigorously disinfected after each patient contact. Figure 1 shows the schematic representation of the outbreak management concept we implemented in order to combat the local EKC outbreak.

### Ethics statement

The ethics committee of Saarland/Germany approved this study (Nr. 18/21). The study was conducted in accordance with the Declaration of Helsinki after obtaining written informed consent from all patients.

### Statistical analysis

Continuous variables are expressed as median value [range], categorical variables in absolute number, and percentage. The Chi-square or Fischer’s exact test was used for analysis of categorical variables, while Kruskal–Wallis test and Wilcoxon rank sum were used for continuous variable analysis of the non-parametrical data. Exponential curve fit analysis was used to determine the intrinsic growth rate of EKC cases. All statistical tests were two-sided and a *p* value of < 0.05 was considered statistically significant. Bonferroni method was used to adjust for multiple testing. All data were analyzed using SPSS v25.0 (IBM, Ehningen, Germany).

## Results

Between 20th August 2018 and 4th November 2018, 120 patients with symptoms and signs of EKC were examined. The median age was 58 [1–92] years*.* Among all included patients, 61 (50.8%) were female. The median visual acuity was 0.6 [0.02–1] for the right eye and 0.6 [0.02–1.25] for the left eye (*p* = 0.65).

### Outbreak investigation

**Fig. 2:**
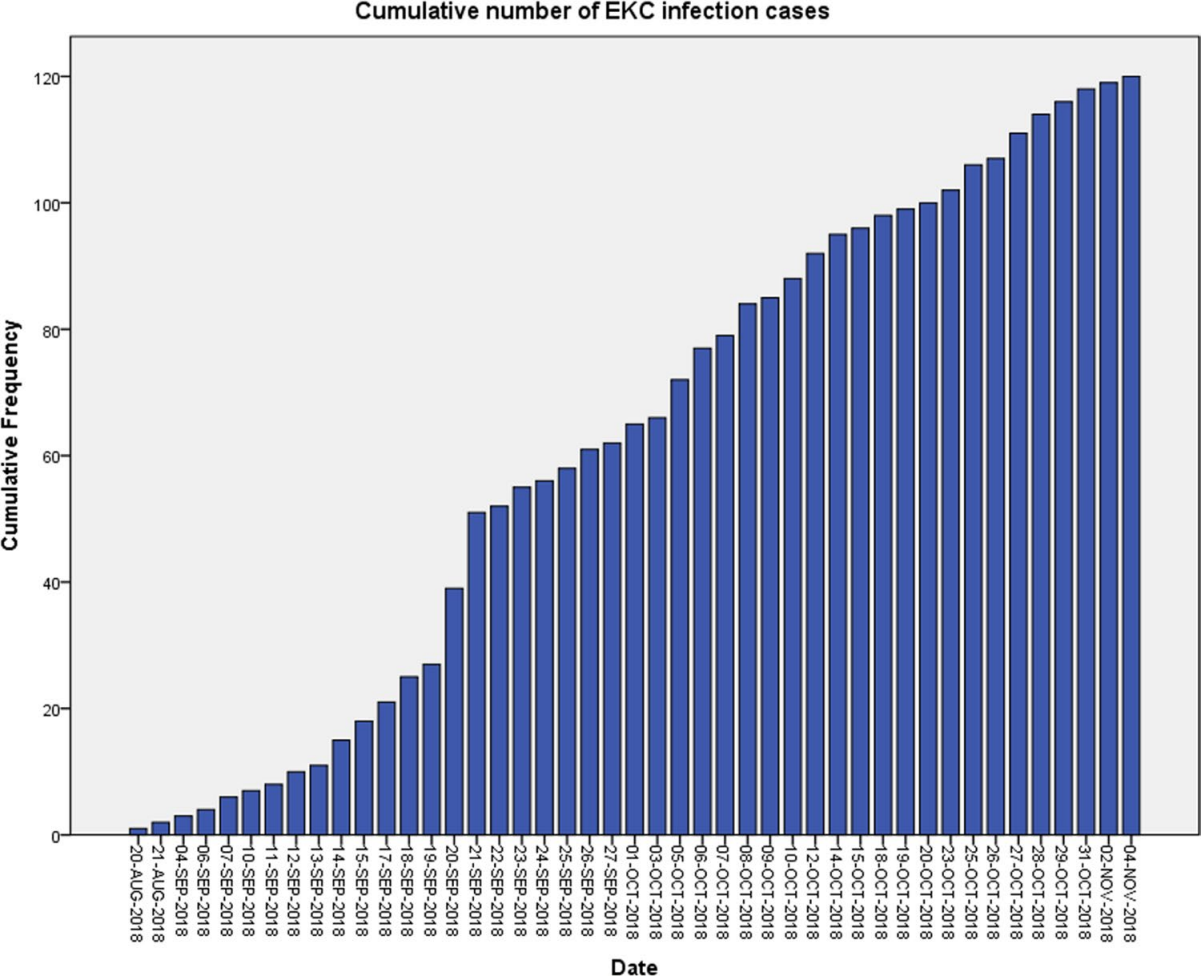
Cumulative number of suspected EKC infection cases in our department in the period from 20th August 2018 to 4th November 2018

The first suspected EKC case in our department was registered on 20th August 2018. This date marked the beginning of the EKC outbreak. The outbreak lasted 77 days (20th August 2018 to 4th November 2018) and affected a total of 120 patients. This corresponds to a mean of 1.5 patients per outbreak day. The cumulative number of cases with suspected EKC in our clinic is presented in Fig. 2.

**Fig. 3:**
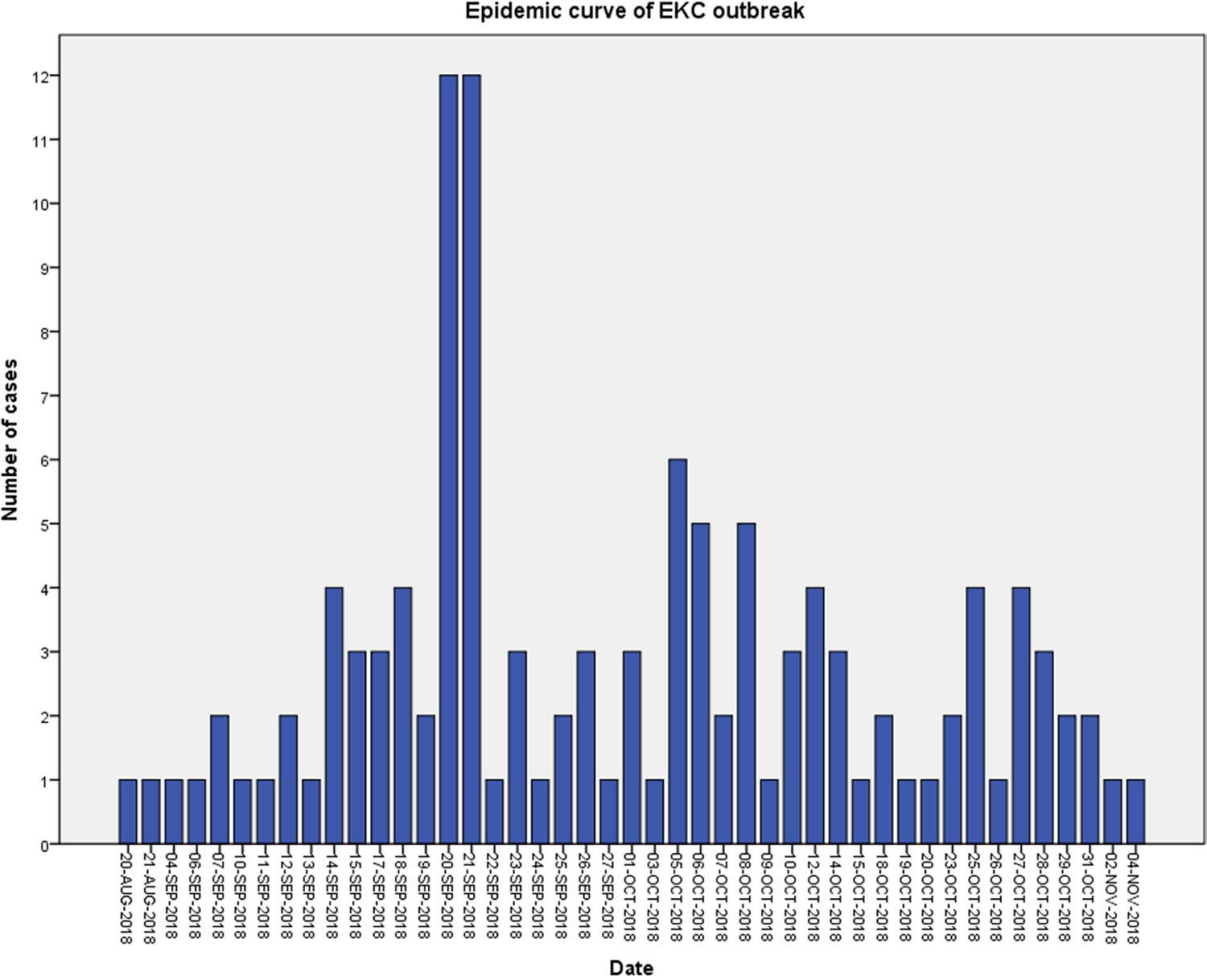
Epidemic curve of outbreak of EKC in Homburg/Saar, Germany, in the period from 20th August 2018 to 4th November 2018

The highest number (12 patients/day) of suspected cases of EKC was registered on both 20th and 21st September 2018. On the last day of the outbreak (4th November 2018), only 1 suspected case was registered. The epidemic curve of the outbreak of EKC is presented in Fig. 3.

**Fig. 4:**
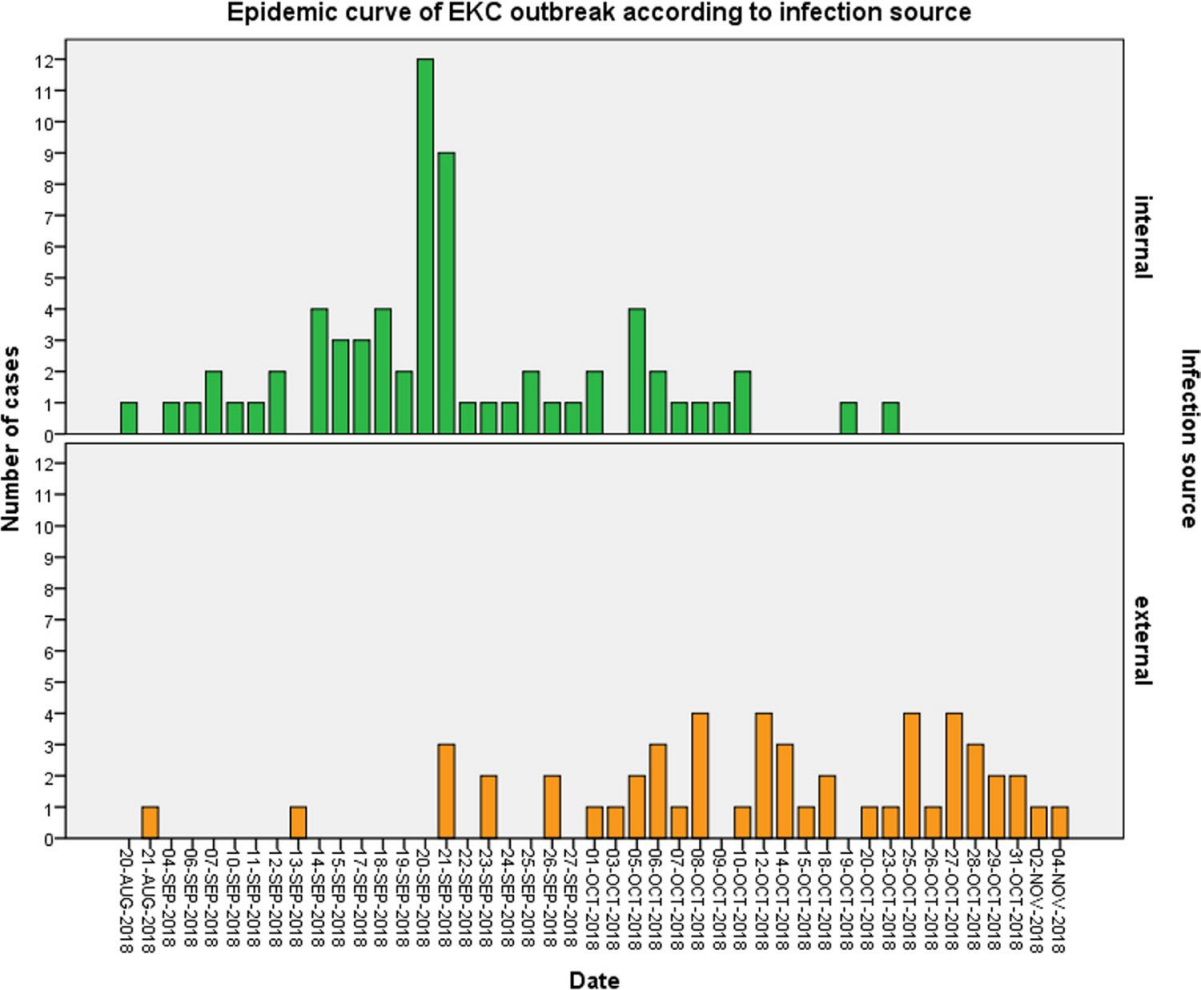
Epidemic curves of EKC outbreak according to infection source

Furthermore, we examined the new daily cases of suspected EKC according to the infection source. Epidemic curves of the EKC outbreak are presented in Fig. 4. The first external case was registered on 21st August 2018, while the first internal case 1 day earlier, on 20th August 2018. The peak number of internal cases (12 cases) was registered on 20th September 2018, while the highest number of external cases (4 cases) was on 8th October 2018. The epidemic duration was 65 days concerning internal cases and 76 days concerning external cases. The median duration from onset of symptoms to clinical presentation was 4.5 [1–12] days for internal cases in comparison to 3.0 [2–10] days for external cases (*p* value = 0.65). As such, the internal epidemic started earlier, reached higher peak numbers and lasted less than the external outbreak (Fig. 4).

### Clinical presentation

The clinical manifestations of EKC were collected in all 120 cases. In 67/120 (55.8%) cases, the right eye was the first eye to be affected by EKC. The second eye was affected in 62/120 (51.7%) cases. The median time span between the affected eyes was 4 [1–12] days. In 34/62 (54.8%) cases, the EKC was bilateral at presentation.

The clinical manifestations according to the infection source are presented in Table 1. Chemosis was significantly more frequent in internal cases compared to external cases (44/68, 64.7%, and 13/52, 25.0%, χ^2^ = 17.34, *p* value = 0.01). Patients with internal infection had a significantly higher proportion of conjunctival follicles compared to patients with external infection (46/68, 67.6%, and 18/52, 34.6%, χ^2^ = 11.66, *p* value = 0.01).

**Table 1:** Clinical presentation according to infection source (internal vs. external)

Clinical presentation	Infection source		*p* value
	Internal *N* = 68	External *N* = 52	
Chemosis	44 (64.7%)	13 (25.0%)	0.01
Conjunctival congestion	60 (88.2%)	37 (71.2%)	0.04
Conjunctival follicles	46 (67.6%)	18 (34.6%)	0.01
Conjunctival petechiae	5 (7.4%)	5 (9.6%)	0.60
Corneal epithelial punctate	17 (25.0%)	9 (17.3%)	0.40
Plica/caruncle swelling	47 (69.1%)	26 (50.0%)	0.06
Pseudomembranes	10 (14.7%)	5 (9.6%)	0.40
Corneal erosion	4 (5.9%)	4 (7.7%)	0.66
Corneal epithelial infiltration	14 (20.6%)	10 (19.2%)	0.86
Eye discharge	8 (11.8%)	10 (19.2%)	0.25

### Virological screening

Conjunctival swabs were collected in 100/120 (83.3%) cases. All the external cases did not have a performed EKC swab before presentation in our clinic. Among all swabs, 63/100 (63.0%) were positive, all (63/63, 100%) for human adenovirus. A subgroup of 5 randomly selected samples was sequenced, and adenovirus serotype 8 was detected.

### Evaluation of the outbreak management concept

In order to evaluate the results of our outbreak management strategy, the EKC cases per day of the outbreak was calculated. As such, in phase 1, a total of 39 EKC cases were registered. This corresponded to a mean of 1.2 ± 0.4 cases per outbreak day. In phase 2, 24 EKC cases were diagnosed, and therefore, the mean was 2.2 ± 0.9 cases per outbreak day. Phase 3 included only internal EKC cases, as primarily influenced by our EKC outbreak management concept and displayed a mean of 0.6 ± 0.1 cases per outbreak day. Regarding phases 1 and 2 together, the mean EKC cases per outbreak day was 1.4 ± 0.3. Thus, the mean EKC cases per outbreak day was significantly lower in phase 3 compared to phases 1 and 2 combined (*p* value = 0.001). The reduction in number of EKC cases per outbreak day corresponded to a Cohen’s *d* of − 0.415 (95% CI [− 0.93, − 0.10]).

The second outcome of interest in our analysis was the basic reproductive number in internal cases before and after EKC outbreak strategy implementation. As Fig. 3 shows, this corresponded to 2nd October 2018. Using the maximal incubation time of 12 days, we obtained a maximal basic reproduction number for phases 1 and 2 (R01) combined of 1.18. The maximal basic reproduction number for phase 3 (R02) was 0.52, corresponding to a reduction by a factor of 2.2.

## Discussion

We report a large EKC outbreak that took place in our ophthalmological clinic between 20th August and 4th November 2018. The study included 120 patients with suspected EKC and accumulated data over 77 outbreak days.

The epidemic curve of our EKC outbreak points mainly to a scattered type of infection transmission, since cases follow one another over a longer period. The scattered type of transmission in external EKC cases corresponds to a prolonged epidemic duration which summed up to 75 days in this outbreak. In comparison, the internal EKC outbreak lasted only 64 days, and its epidemic curve indicated, by summing up a large number of cases in a few epidemic days, a sudden infection onset with a possible common infection source. Therefore, we decided to concentrate our hygienical disease control strategies primarily on the internal EKC cases.

The prompt recognition of an ongoing EKC outbreak and rapid implementation of our hygienic outbreak management concept resulted in a shorter outbreak duration compared to other reported healthcare-related EKC outbreaks [14]. Consistently, the duration of EKC outbreak was significantly shorter in internal cases (65 days), where the outbreak management concept was implemented, compared to external cases (76 days). The implementation of our hygienic management concept started earlier compared to the published literature and as such possibly contributing to a greater reduction in EKC cases [15, 16].

Our EKC outbreak management concept followed the international and national recommendations and was specifically adapted to our epidemiological situation [17, 18]. We elaborated and implemented an outbreak management concept that incorporated patients, their families, and medical and non-medical staff in our department. The rapid and consistent implementation of the hygienically oriented measures helped flattening the epidemic curve of the EKC outbreak. The strongest effects of these interventions were seen in the reduction of the number of internal EKC cases per outbreak day.

In order to quantify the effectiveness of our outbreak management concept, we used, in addition to number of cases per outbreak day, the basic reproduction number. The basic reproduction number for susceptible, exposed, infectious, and recovered (SEIR) epidemiological models allows the quantification of different disease control strategies, such as our hygienic outbreak management concept [19]. To our knowledge, this is the first analysis of an EKC outbreak that used the basic reproduction number to assess the efficacy of an outbreak management concept. Results of our analysis showed that the implementation of our hygienic management concept decreased significantly the EKC cases per outbreak day and also the basic reproduction number by a factor of 2.2. As such, the average number of secondary cases produced by one patient with EKC was two times lower after the implementation of our outbreak concept. The use of a quantifiable measure, such as the basic reproductive number, allows comparison between different EKC hygienical concepts and might contribute to a better standardized clinical practice in EKC.

Our study has some limitations. Firstly, we collected the data retrospectively, and therefore, selection and recall biases might be present. Secondly, virological conjunctival swabs could not be obtained in all cases. In response to this limitation, the proportion of collected swabs to total number of cases was higher in our study compared to previous conducted one [16]. Thirdly, we were not able to collect data on viral load. Differences in viral loads could explain the more severe clinical presentation in internal cases compared to external ones and, as such, could confound the results on outbreak duration and efficacy of our EKC management concept. Furthermore, different adenovirus serotypes could have influenced some of the results. Serotyping was performed in our study randomly in only 5 samples and confirmed adenovirus serotype 8. Thus, it is plausible that the outbreak was driven by this not unusual serotype, but the introduction of another serotype cannot completely be excluded.

Nevertheless, our study has its strong points. The EKC outbreak was reported in conformity with the ORION statement [12]. It enrolled a relatively large cohort of suspected EKC cases. Conjunctival swabs were collected in a high number of cases. This assured a rapid confirmation of suspected EKC cases and had a direct influence on EKC outbreak recognition and cessation. The EKC outbreak was promptly recognized, and a series of hygienically oriented interventions was successfully applied to further reduce transmission. The use of an EKC outbreak management concept that incorporated mainly general hygienical measures (easily applied in different settings), characteristic target population, and common EKC outbreak setting (ophthalmological department) might assure higher degree of generalizability of our results. The quantification of effectiveness using the basic reproductive number before and after EKC management implementation will allow comparison between different EKC management concepts and different hospitals. Our results are in line with current evidence, suggesting efficacy of hygienical measures in reducing EKC spread [8, 11].

In conclusion, we could demonstrate that implementing our outbreak management concept resulted in a significant reduction of EKC cases, thus potentially avoiding complications and unnecessary health-related costs.

## Data Availability

Data is available at request from the first author.
